# Safety and Efficacy of Stosstherapy in Nutritional Rickets

**DOI:** 10.4274/jcrpe.3557

**Published:** 2017-03-01

**Authors:** Daipayan Chatterjee, Mathad K. S. Swamy, Vikas Gupta, Vasu Sharma, Akshat Sharma, Krishti Chatterjee

**Affiliations:** 1 Vardhaman Mahavir Medical College, Department of Orthopedics, New Delhi, India

**Keywords:** Stosstherapy, Nutritional rickets, Vitamin D, Alkaline phosphatase

## Abstract

**Objective:**

Stosstherapy has been used since early 19^th^ century for treating nutritional rickets. However, there are no clear cut guidelines for the biochemical monitoring of this treatment. Repeated blood tests at short intervals increase the cost of therapy and noncompliance.

**Methods:**

A prospective study was conducted on 191 cases of nutritional rickets below 10 years of age to evaluate the effectivity of stosstherapy. All cases were treated with a single intramuscular injection of vitamin D (600.000 IU) along with oral calcium (50 mg/kg) and vitamin D (400 IU per day) until radiological resolution. Dietary modifications and adequate sunlight exposure were also recommended.

**Results:**

The mean age of presentation was 2 years 9 months. Mean sunlight exposure was 17 minutes/week with 90% having low sunlight exposure (<30 minutes/week). Prolonged breast feeding (>6 months) was found in 93.7% of the cases. With treatment, the clinical features started resolving by 1 month with complete resolution of most of the features over a period of 1 year. By 6 months, all the study subjects had complete radiological resolution. Serum levels of calcium and alkaline phosphatase (ALP) were restored by 6 months in most cases while phosphate and vitamin D levels normalized by 6 weeks.

**Conclusion:**

Stosstherapy is a safe, cheap and effective method of treating nutritional rickets. Biochemical tests at initial presentation followed by vitamin D assay at 6 weeks and calcium, phosphate and ALP assays at 6 months is recommended in the monitoring of these patients. For regular monitoring, only ALP assay is recommended, provided one abstains from repeat injection of vitamin D based on high ALP levels.

**WHAT IS ALREADY KNOWN ON THIS TOPIC?**

Stosstherapy may be used for treating nutritional rickets.

**WHAT THIS STUDY ADDS?**

Single injection is sufficient enough for treating nutritional rickets. Alkaline phosphatase may be used as a secondary marker for monitoring. Vitamin D assay at 6 weeks and calcium, phosphate and alkaline phosphatase assay at 6 months is recommended to monitor the biochemical improvement.

## INTRODUCTION

Rickets is a metabolic bone disease characterized by deficiency of vitamin D and/or dietary calcium leading to impaired mineralization of newly formed bone matrix before epiphyseal closure (1). Nutritional rickets has undergone a recent surge in frequency during the last decade especially in developed countries where it was thought to have been eradicated (2). In developing countries, rickets has been ranked among the five most prevalent childhood diseases (3).

Nutritional rickets can be treated by various regimes ranging from daily or weekly oral therapy to single high-dose (stosstherapy) oral or intramuscular therapy. The advantage of intramuscular high-dose therapy over other regimes is avoidance of daily dose, thereby increasing patient compliance and decreasing cost of therapy. On the other hand, the main disadvantages are injection site morbidity and potential complication of vitamin D toxicity. Stosstherapy has been used since early 19th century for treating rickets (4). However, there are no clear cut guidelines as to which biochemical tests are needed and at what interval these tests are to be repeated for monitoring the effect of this treatment. Repeated blood tests at short intervals not only increase the cost of therapy but also increase noncompliance.

This study not only aims to evaluate the effectivity of stosstherapy in nutritional rickets but also addresses the above-mentioned problems.

## METHODS

A longitudinal prospective study was conducted over a 3-year period from September 2011 to September 2014 at Vardhaman Mahavir Medical College and Safdarjung Hospital (Central Institute of Orthopedics, New Delhi, India). Out of 212 cases of rickets attending the outpatient department, 196 untreated cases of nutritional rickets of ages below 10 years were selected on the basis of clinical, biochemical, and radiological features. Out of these cases, 5 were lost to follow-up. Thus, 191 patients were enrolled for the study after written informed consent was obtained from the parents. Cases with a history of prematurity, renal or hepatic disease, intestinal malabsorption, tumor, chronic diseases including tuberculosis and diseases of the skeletal system were excluded. Pretreatment biochemical investigations included estimation of serum calcium, phosphate, alkaline phosphatase (ALP), 25-hydroxyvitamin D (calcidiol), parathyroid hormone (PTH) levels along with urinary calcium and phosphate excretion. Rickets cases with normal PTH (hypophosphatemic rickets), phosphaturia (renal rickets-renal tubular acidosis type 1 and 2 and hypophosphatemic rickets), and high levels of calcidiol (vitamin D-dependent rickets types 1 and 2) were excluded. The cases so segregated were evaluated by taking a detailed history which included enquiry regarding average weekly sunlight exposure, duration of breast feeding, and average daily cow milk consumption in children more than 6 months (measured by determining the volume of the container used by the child to drink milk). Antero-posterior views of bilateral wrist and knee joints were used for radiological evaluation.

The selected cases were treated with a single high-dose (600.000 IU) intramuscular injection of cholecalciferol (Arachitol 6L, Abbott India) along with oral calcium (50 mg/kg) and vitamin D (400 IU per day) until radiological resolution. Dietary modifications (egg, milk, fish added to the diet) and adequate sunlight exposure (30 minutes/weekly) were also advised.

Calcidiol and PTH were estimated by chemiluminescent microparticle immunoassay (CMIA; Abbott Architect Plus i1000SR). Intra- and inter-assay coefficients of variation (CVs) for calcidiol were 1.4-3.7% and 2.7-4.6%, respectively. Calcium, phosphate, and ALP were estimated by spectrophotometry using arsenazo-3, ammonium molybdate, and para-nitrophenyl phosphate as reagents, respectively (Vitros 5.1 FS analyzer- Johnson and Johnson). Ethical clearance was obtained for the study from the hospital ethical clearance committee.

Follow-up was done at 3 weeks, 6 weeks, 3 months, 6 months, 9 months, and 1 year by estimation of serum calcidiol, calcium, phosphate, ALP levels and by radiographs of both wrist and knee joints (antero-posterior views bilaterally). Radiological resolution was quantified using Thacher’s 10-point scoring system (5) where a score of ‘0’ was considered to be complete radiological resolution. The cases were clinically examined by the corresponding author at all visits. All the radiographic films were reviewed and scored by the same radiologist (who was blinded to the study) and the corresponding author separately. All the cases were subjected to abdominal ultrasonography prior to treatment and at 6 months and 1 year posttreatment to check for nephrocalcinosis.

Pre- and post-treatment clinical, biochemical, and radiological parameters were compared and analyzed statistically by using paired t-test for quantitative data and chi-square test for qualitative data with the help of SPSS 20 (IBM Corp. Released 2011. IBM SPSS Statistics for Windows, Version 20.0. Armonk, NY: IBM Corp). Data pertaining to sunlight exposure, initial vitamin D levels, and duration of breast feeding were compared.

## RESULTS

One-hundred and ninety-one cases (85 male, 106 female) were enrolled in the study. The mean age of presentation was 2 years 9 months (range 6 months-9 years). Mean sunlight exposure was 17 minutes/week (range 0 -2 hours/week) with 51% (97 cases) having no sunlight exposure and 90% (172 cases) having sunlight exposure of less than 30 minutes/week. Those exposed to sunlight were partially dressed (dress covering body and extremities up to mid-arm and mid-thigh) during the exposure. In 93.7% (179 cases) of the cases, there was a history of prolonged breast feeding (more than 6 months). Mean duration of breast feeding was 1 year 8 months (range 0-42 months). Average daily cow milk consumption was 60 mL (range 0-250 mL) with 48% (92 cases) having no milk consumption and 96.3% (184 cases) consuming 200 mL or less.

Table 1 enumerates the frequency and resolution of clinical features during the follow-up period. Swollen wrist and ankle was the most common clinical feature followed by angular deformity of the lower limb. Clinical features started resolving by 1 month. The earliest sign of clinical improvement was increase in physical activity of the child as reported by the parents.

Biochemical findings are presented in Table 2. The mean serum calcium at initial presentation was 8.8±0.6 mg/dL with 87% having hypocalcemia. Similarly, 67% cases presented with hypophosphatemia with a mean of 3.5±0.8 mg/dL. High ALP levels were noted in 91% cases at initial presentation with a mean of 1315±914 IU/L. By 6 months of treatment, the majority of the cases had achieved normal serum calcium (91%), phosphate (100%), and ALP (87%) levels. On the other hand, 98% had hypovitaminosis at presentation with a mean serum level of 13±8 ng/mL. Normal vitamin D levels were restored in the majority (95%) of the cases by 6 weeks and in all the cases by 3 months. The change of mean serum calcium level from the previous visit was found to be statistically significant at every visit in the first 6 months of treatment (Table 3). Apart from the change of mean serum phosphate between the 6th and 9th months of treatment and the change in mean serum vitamin D level between the 3^th^ and 6^th^ months of the follow-up, all the changes in mean serum phosphate and vitamin D levels from the level at the previous visit were found to be statistically significant. Since the normal range of ALP is an age- and sex-dependent variable, data in Table 2 pertaining to ALP is adjusted for age and sex of the cases and classified as hyper, normal and hypo as per Turan et al (6) Vitamin D levels were defined as deficiency, insufficiency, sufficiency, excess, and toxicity as per the United States Endocrine Society classification (5,7).

The mean radiological score at initial presentation was 6.9±3.1 (range, 1-10) with 45% (86 cases) having a score of 10. All the cases showed a healing line for rickets by 6 weeks. Around 47% (90 cases) had complete radiological resolution by 3 months. By 6 months, all the study subjects had complete radiological resolution. Serial abdominal ultrasonography did not reveal nephrocalcinosis in any of the cases.

## DISCUSSION

The word “stosstherapy” has been derived from the German word “stossen” meaning “to push”. It involves the use of large doses of vitamin D to treat nutritional rickets. This treatment approach is based on the fact that vitamin D is efficiently stored in adipose tissue and muscles after a single large dose, following which continued conversion to the active metabolite 1,25-dihydroxy vitamin D helps to heal rickets. The main advantages of intramuscular stosstherapy over daily or weekly regimes or oral stosstherapy are compliance and cost. Only a single injection is to be administered which ensures good compliance. Each vial of ‘Arachitol 6L’ costs around 0.37 USD (United States Dollar), while the cost of other regimes is more than 10 times the cost of intramuscular stosstherapy.

Children with nutritional rickets were noted to have low sunlight exposure (90%), prolonged breast feeding (93.7%), and low consumption of cow milk (96.3%). There is very little research available to determine exactly how much sun exposure is necessary to maintain adequate vitamin D levels. Due to variations in age, skin color, latitude, time of day, and time of year, it is impractical to provide prescriptive advice to the population as a whole (8). Based on available research, it has been estimated that exposure of 40% of the body for around 30 minutes/week will result in generation of 1000 IU of vitamin D per day (9). Human milk has a low vitamin D (20 IU/liter) content (10,11). Assuming an average consumption of 750 mL/day, exclusive breast feeding provides only 11-38 IU/day of vitamin D, which is well below the recommended dietary allowance for an infant (12). Hence, as the vitamin D reserves are depleted (which takes around 6 months provided the mother was not vitamin D-deficient during pregnancy or postpartum), the infant starts developing signs of deficiency unless vitamin D is supplemented. In our study, we found a negative correlation between vitamin D at presentation and the duration of breast-feeding and a positive correlation between sunlight exposure and initial calcidiol level implying that prolonged breast-feeding (>6 months) and low sunlight exposure (<30 minutes/week) lead to a decrease in vitamin D levels thus increasing the chances of rickets. Hence, we recommend exclusive breast feeding up to 6 months and sunlight exposure of at least 30 mins/week.

**Figure 1:**
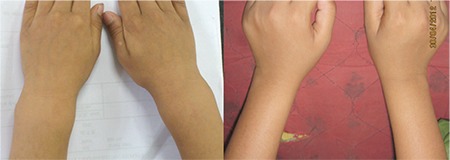
Resolution of swollen wrist at 1-year follow-up

**Figure 2:**
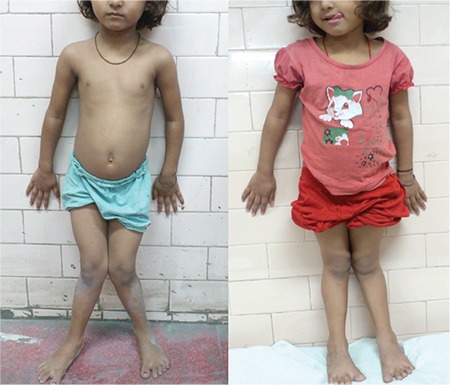
Reduction in the genu valgum deformity in a 9-year-old child at 1-year follow-up

**Figure 3:**
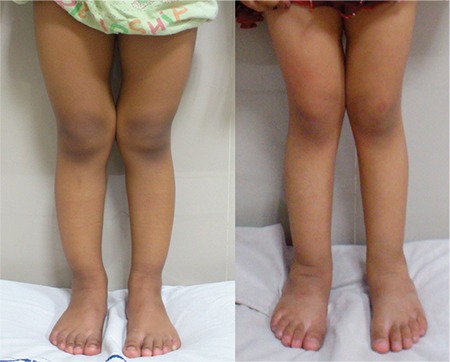
Resolution of varus angular deformity in the lower limb at 1-year follow-up

Swollen wrist and ankle were the most common clinical features (95%). They started improving by 6 weeks and resolved completely by 1 year (Figure 1). Angular deformity of the lower limb was the second most common presenting feature. In the majority of the cases, the varus deformity resolved by 1 year, while the remaining in the remaining minority, this finding neither improved nor deteriorated. It was noted that most of those who improved had mild deformities. Genu valgum was observed in children older than 4 years. Stosstherapy reduced the quantum of angulation in both genu valgum (Figure 2) and windswept deformity but failed to achieve complete resolution. These observations showed that the milder the deformity and the younger the child at initiation of treatment, the more the chances of resolution. The possible explanation for this observation is that with proper metabolic control, the lower limb bones resume a normal growth pattern. The initial varus deformity present in young children over time gradually changed to neutral or slight valgus (Figure 3). Other clinical signs such as frontal bossing (52%), pot belly (38%), rachitic rosary (11%), and Harrison sulcus (5%) all started improving by the 6^th^ week of treatment and achieved complete resolution by 9 months. Enamel hypoplasia (26%), repeated fractures (7%), and short stature (9%) were a few other less commonly observed features which also improved during follow-up. Thus, it can be concluded that stosstherapy led to clinical improvement in all the cases with complete resolution of most of the features over a period of 1 year. Our results are supported by studies conducted at Greater New Haven by DeLucia et al (13) on 43 cases and in Western Nigeria (14) on 26 cases with nutritional rickets.

**Figure 4:**
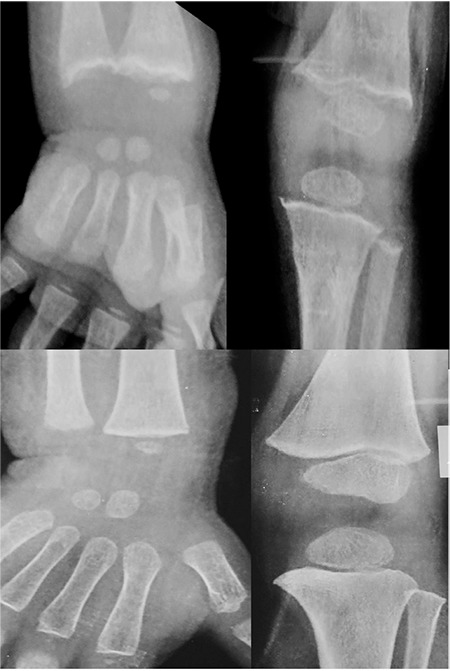
Radiogram showing healing at 6-month follow-up

Ozkan (15) has aptly enumerated the radiological changes noted during rickets. The earliest radiological findings are observed at the distal ulnar region in infants and the knee in older children. Expansion of the metaphysis (splaying), irregularity of the metaphyseal margin (fraying) giving it a brush-like appearance, cupping, and general osteopenia are the typical radiological findings in cases of nutritional rickets. In our study also, we came across these characteristic radiological changes. Cupping, splaying, and fraying noted in the initial radiographs were found to disappear gradually with treatment. Complete radiological resolution was achieved in all the cases by 6 months (Figure 4).

By 6 months, the majority of the cases had also achieved normal serum calcium (91%), phosphate (100%), and ALP (87%) levels. On the other hand, normal vitamin D levels were restored in the majority of the cases (95%) by 6 weeks and in all cases by 3 months. It was noted that there is no requirement for frequent biochemical tests during follow-up visits after stosstherapy. Thus, biochemical tests at initial presentation followed by a vitamin D assay at 6 weeks and calcium, phosphate, and ALP assays at 6 months are sufficient to monitor the biochemical improvement with treatment. This will not only reduce the cost of therapy but also help improve patient compliance by decreasing the number of needle pricks.

Hypercalcemia (2%), hyperphosphatemia (1%), and hypervitaminosis (5%) were noted in only a few cases during follow-up, but none of the cases were symptomatic, further ensuring the safety of the regime. Cesur et al (16) reported resolution of nutritional rickets with mega doses of vitamin D. Asymptomatic hypercalcemia was detected in 6 of their patients.

In the first year of follow-up, the mean ALP level at each visit was found to differ significantly from the level at the previous visit, a finding which implies that the ALP assay, if repeated at every visit, can monitor the biochemical improvement during follow-up. Thus, if the physician wants to monitor the biochemical improvement regularly, only ALP assay at follow-up visits is recommended as it is a reliable yet cost-effective alternative to the costly vitamin D assay. However, repeat injection of high-dose vitamin D should not be given based on high ALP levels during the follow-up period since ALP takes a long time to be restored (6-9 months). Injection of mega-dose vitamin D should only be repeated if the healing line of rickets does not appear in the radiograph of the wrist by 6 weeks and the patient’s diagnosis is confirmed to be nutritional rickets (17).

Our findings indicate that stosstherapy leads to clinical, biochemical, and radiological resolution in nutritional rickets. A similar conclusion was also reported by Shah and Finberg (18) and Cesur et al (16). Thus, it can be concluded that stosstherapy is a safe, cheap yet effective method of treating nutritional rickets. A single injection of mega-dose vitamin D is sufficient for treating nutritional rickets. Stosstherapy also requires less frequent monitoring. Biochemical tests at initial presentation followed by a vitamin D assay at 6 weeks and calcium, phosphate, and ALP assays at 6 months is recommended to monitor the biochemical improvement with treatment. This will further reduce the cost of therapy and improve compliance of the patient. However, if the physician wants to monitor biochemical improvement regularly, serial assays of ALP only are recommended, provided one abstains from repeating injections of mega dose of vitamin D based on high ALP levels.

## Figures and Tables

**Table 1:**
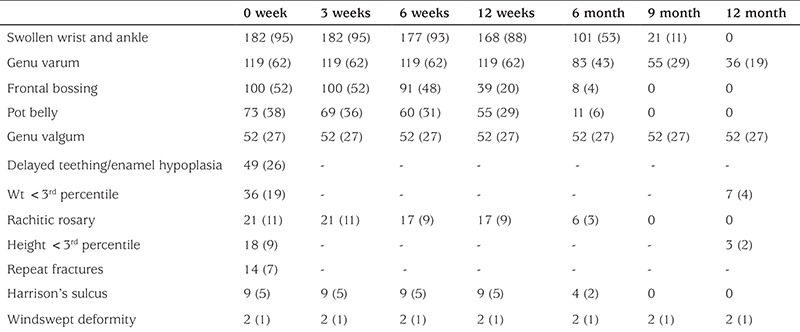
Frequency and resolution of clinical features during a one-year follow-up period

**Table 2:**
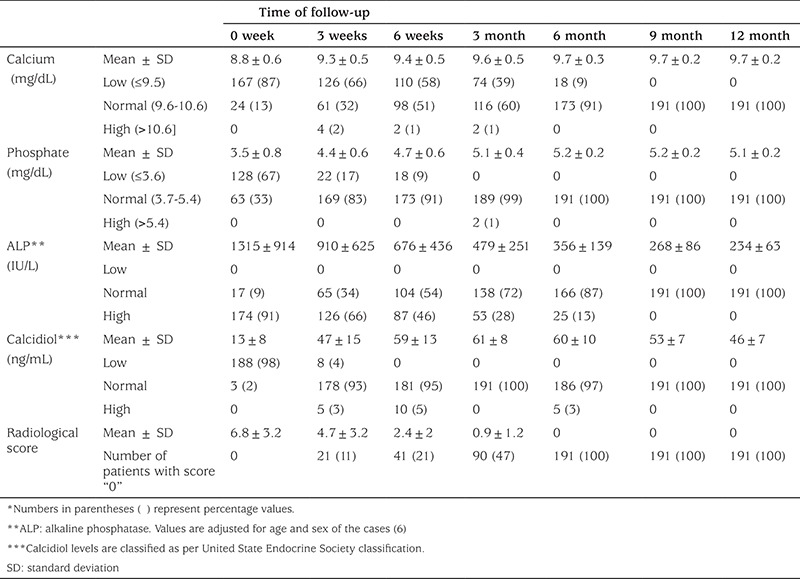
Biochemical and radiological changes during follow-up

**Table 3:**
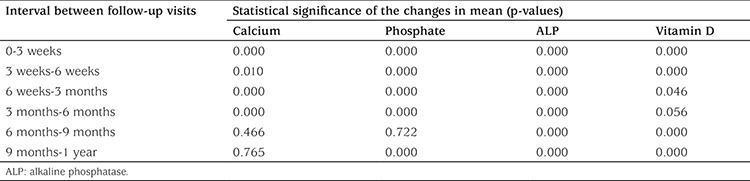
Statistical significance of changes in mean serum levels of calcium, phosphate, alkaline phosphatase, and calcidiol between follow-up visits
